# An Attempt to Evaluate Selected Aspects of “Bone–Fat Axis” Function in Healthy Individuals and Patients With Pancreatic Cancer

**DOI:** 10.1097/MD.0000000000001303

**Published:** 2015-08-14

**Authors:** Wojciech Blogowski, Katarzyna Dolegowska, Anna Deskur, Barbara Dolegowska, Teresa Starzyńska

**Affiliations:** From the Department of Internal Medicine, University of Zielona Góra, Zielona Góra, Poland (WB); Department of Laboratory Diagnostics and Molecular Medicine, Pomeranian Medical University, Szczecin, Poland (KD); Department of Gastroenterology, Pomeranian Medical University, Szczecin, Poland (AD); Department of Microbiology and Immunological Diagnostics, Pomeranian Medical University in Szczecin, Szczecin, Poland (BD); and Department of Gastroenterology and Internal Medicine, Warsaw Medical University, Warsaw, Poland (TS).

## Abstract

Recently, much attention has been paid to a potential biochemical cross-talk between the metabolism of the adipose tissue (AT) and bone (marrow), termed “bone–fat axis.” We hypothesized that selected substances, participating in this “dialog,” are associated with body mass and peripheral trafficking of bone marrow-derived stem cells (BMSCs) in both healthy individuals and patients with obesity-associated malignancies such as pancreatic adenocarcinoma.

We performed an analysis of the systemic levels of selected substances involved in the regulation of bone (marrow) homeostasis (parathormone, calcitonin, osteopontin, osteonectin, stem cell factor [SCF], and fibroblast growth factor-23) in 35 generally healthy volunteers and 35 patients with pancreatic cancer. Results were correlated with the absolute number of circulating BMSCs and body mass values. Additionally, subcutaneous and visceral/omental AT levels of the aforementioned molecules were analyzed in lean and overweight/obese individuals.

Intensified steady-state trafficking of only Lin-CD45 + CD133 + hematopoietic stem/progenitor cells was observed in overweight/obese individuals and this was associated with BMI values and elevated levels of both osteonectin and SCF, which also correlated with BMI. In comparison to healthy individuals, patients with cancer had significantly higher osteopontin levels and lower values of both osteonectin and osteonectin/osteopontin ratio. While no significant correlation was observed between BMI and the number of circulating BMSCs in patients with cancer, peripheral trafficking of CD34 + KDR + CD31 + CD45-endothelial progenitor cells and CD105 + STRO-1 + CD45-mesenchymal stem cells was associated with the osteonectin/osteopontin ratio, which also correlated with BMI (r = 0.52; *P* < 0.05). AT levels of the examined substances were similar to those measured in the plasma, except for osteonectin, which was about 10 times lower.

Our study highlights the potential role of osteonectin, osteopontin, and SCF as communication signals between the bone (marrow) and AT in both healthy individuals and patients with pancreatic cancer. We postulate that these molecules may be overlooked biochemical players linking body mass and BMSCs with obesity-associated cancer development and/or progression in humans.

## Abstract 2

Supplemental Digital Content is available in the text

## INTRODUCTION

Adipose tissue (AT) is a very dynamic organ that participates in the regulation of multiple (patho)physiological processes, including energy balance, hormonal homeostasis, and/or aging. Excessive accumulation of AT, defined as obesity, remains one of the most challenging public health problems in several countries, as it is associated with the development of systemic metabolic abnormalities that affect function and metabolism of numerous organs, inevitably leading to their impairment.^1^ According to recent trends, obesity is also becoming a very important preventable risk factor of various gastrointestinal cancers and, more importantly, obese patients have significantly poorer outcomes following the development of these neoplasms.^2–4^ Specifically, they tend to present a younger age of disease onset, multifocal neoplasms, poorer short-term recovery, increased rate of perioperative complications following cancer surgery, higher rates of cancer recurrence, shorter overall survival, and elevated risk of developing other obesity-associated malignancies.^5–11^ While it is generally attributed to intensified/abnormal signaling of insulin/insulin-like growth factors, altered profile of adipokines, and/or modulated systemic immune/cytokine balance, the exact molecular mechanisms responsible for this link in humans still remain poorly understood and require further evaluation.^2,4,12,13^

In recent years, much attention has been paid to a potential systemic dialog between the AT and bone (marrow) metabolism, termed the “bone–fat axis”.^14–18^ This interest arose from multiple clinical observations that revealed several hormonal and drug relationships between these 2 tissues such as the presence of impaired bone quality in obese patients with type 2 diabetes or increased risk of accumulation of (especially visceral) AT and development of osteoporosis among patients on chronic corticosteroid therapy.^19,20^ However, the interest in these interactions between the bone and fat metabolism increased due to recent molecular findings demonstrating that both tissues have identical embryonic origin, regulate energy metabolism, are important targets of action of similar growth factors and/or hormonal agents, and share biochemical and structural features that create suitable “niches” for various stem cells.^14,15,21^

This “cross-talk” between the AT and bone (marrow) metabolism seems to be of importance in physiological conditions, but little is known about its potential role in the development/progression of (gastrointestinal) malignancies in humans, even though some studies suggest its significance (reviewed in detail in Ref.^17^). Interestingly, in our recent study, we demonstrated that, in patients with pancreatic cancer, for which obesity is regarded as an important risk factor, an intensified peripheral trafficking of multiple bone marrow-derived stem cell (BMSC) populations is observed.^22^ This phenomenon was associated with systemic levels of complement cascade-derived anaphylatoxins/molecules, which are known to be tightly linked to body mass in humans.^23,24^ Hence, we hypothesized that, in patients with pancreatic cancer, an abnormal function of the “bone–fat axis” occurs both at the systemic and local (tissue) level and may be responsible for the noted phenomenon. Our hypothesis was also supported by the fact that, in patients with neoplasms, the bone and (visceral/omental) AT are regarded as 2 major sites of metastasis from malignancies and may also provide an “attractive” and “vivifying” environment for circulating cancer (stem) cells.^17,25^ Therefore, in this study, we aimed to conduct a translational analysis of selected aspects of a potential biochemical cross-talk between the AT and bone (marrow) metabolism in humans. Specifically, we analyzed several substances involved in the regulation of bone (marrow) metabolism such as parathormone, calcitonin, osteopontin, osteonectin, fibroblast growth factor-23 (FGF-23), and stem cell factor (SCF), which seemed to be the most promising candidates to participate in such a metabolic dialog and also to influence BMSC systemic trafficking. We hypothesized that some of these biochemical substances are associated with body mass and BMSC peripheral trafficking in both healthy individuals and patients with pancreatic cancer and, therefore, may participate in a molecular “cross-talk” in the “bone–fat axis.”

## MATERIALS AND METHODS

A total of 70 individuals with generally good and stable health were included in the study. These patients were divided into a “pancreatic cancer” group (newly diagnosed pancreatic adenocarcinoma, n = 35), and a “control” group (healthy volunteers, n = 35). The final diagnosis of pancreatic adenocarcinoma was based on biopsy specimen analysis. In order to establish disease staging, patients underwent ultrasonography, computed tomography and/or endoscopic ultrasonography, as well as chest X-ray examinations. In the cancer group, 3 patients qualified for surgical removal of the pancreatic tumor (Stage I or II according to the Tumor-Node-Metastasis—TNM classification), 10 patients presented with inoperable, locally advanced disease (Stage III), and 22 patients had distal metastases to the liver or lungs (Stage IV). At the time of their inclusion to the study, none of the patients was on chemotherapy treatment, had received any cytotoxic agents or drugs within the last 12 months before the study, or had presented any clinical signs of an active infectious disease. The general characteristics of these individuals, together with a statistical comparison of their features between the examined groups, are presented in Table 1. Peripheral blood samples (8–10 mL) were collected from these included individuals. Samples were processed according to standard laboratory protocols, and plasma was separated, frozen, and stored at −80°C until further assessment.

**TABLE 1:**
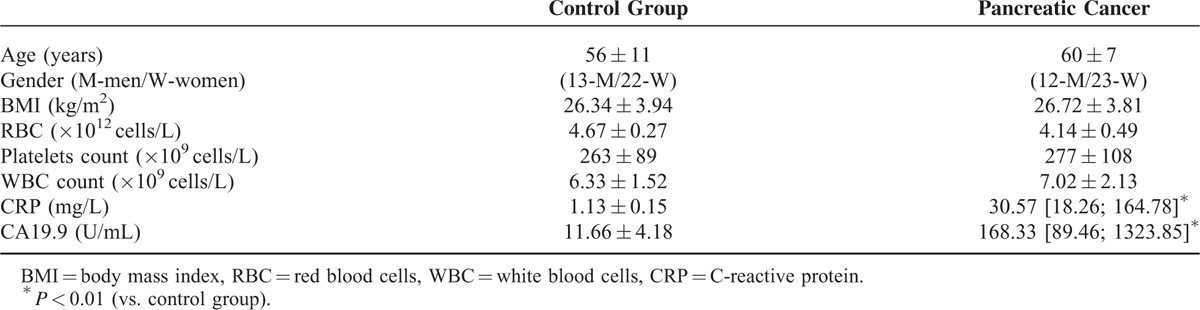
General Characteristic of Individuals Enrolled in the Study (Data Presented as Means ± SD or Median [Interquartile Range])

In order to verify levels of examined molecules in different depots of human AT, plasma and AT samples from generally healthy individuals were used. None of the patients were under hormonal medication, had significantly gained/lost weight within the last 3 months before surgery, nor had any evidence of metabolic diseases other than obesity. Included patients underwent detailed clinical phenotyping, weight was measured in light clothing (rounded up to the nearest 0.1 kg) and height was measured with the subject barefoot using a stadiometer. Afterwards body mass indexes (BMI) were calculated. Patients were classified according to their BMI values as lean (18.5–24.99 kg/m^2^) or overweight/obese (>25 kg/m^2^). General characteristics of these individuals together with statistical comparison of their features between analyzed groups are presented in Supplementary Table 1 http://links.lww.com/MD/A361. AT samples were collected from subcutaneous and omental/visceral depots during elective surgical procedures (laparoscopic cholecystectomy), according to the methods and standards reported previously.^24,26–29^

### Analysis of Systemic and AT-Derived Fat Interstitial Fluid (FIF) Levels of Examined Biochemical Substances

The systemic and AT-derived FIF concentrations of parathormone, calcitonine, osteopontin, osteonectin, FGF-23, and SCF were measured using commercially available, high-sensitivity ELISA kits (Uscn Life Science, Inc., Wuhan, China or TECOmedical AG, Sissach, Switzerland) according to the manufacturer instructions.

### Statistical Methods

Analogically as in our previous studies^29–31^ the Shapiro–Wilk test was used to determine the distribution of the continuous variables analyzed. The Student *t* test was used to compare mean parameter values between the examined groups (for normally distributed variables). For variables that were not normally distributed, the values were log transformed. If a normal distribution was then achieved, these transformed variables were also compared using the Student *t* test. However, if the transformation did not result in a normal distribution, a Mann–Whitney *U* test was performed. Differences between concentrations of analyzed parameters in different AT depots, as well as, between plasma and selected AT fraction were assessed by Wilcoxon paired test. The strength of correlations between examined clinical and biochemical parameters was calculated using Spearman or Pearson rank test, according to the normality of the distribution. Statistical analysis was performed using SPSS statistical analysis software. *P*-values less than 0.05 were considered significant.

The Bioethical Committee of the Pomeranian Medical University in Szczecin approved the study protocol, and patients provided written informed consent before participation.

## RESULTS

### Analysis of Included Individuals

Initial comparison of recruited individuals revealed significantly higher CA19.9 and C-reactive protein (CRP) levels in patients with pancreatic adenocarcinoma when compared to healthy controls (presented in Table 1). No other significant difference was observed between the analyzed groups.

### Comparison of Systemic Levels of the Examined Molecules and Their Associations With the Body Mass

**FIGURE 1:**
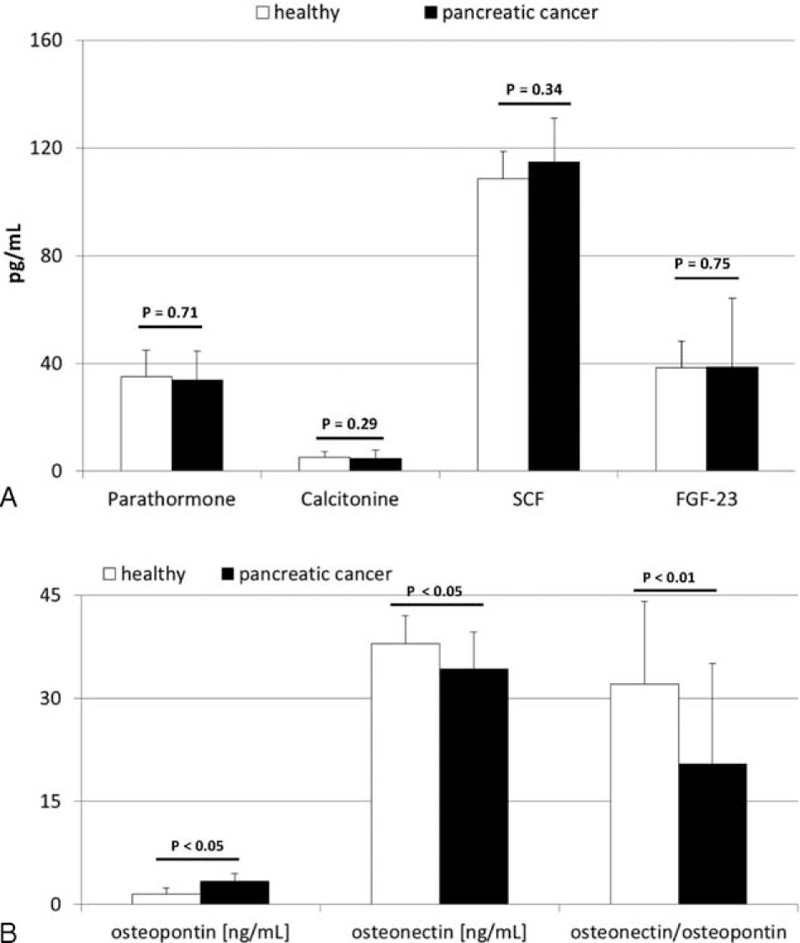
(A and B) Mean concentrations of examined biochemical factors in healthy individuals and patients with pancreatic cancer together with their statistical comparison (data presented as means ± SD). SCF = stem cell factor, FGF-23 = fibroblast growth factor-23, *P* = level of significance.

Mean plasma levels of the examined molecules and their statistical comparison between healthy individuals and patients with pancreatic cancer are depicted in Figure 1A and B. Our analysis demonstrated that, among all examined substances, in comparison to healthy controls, significantly lower osteonectin and higher osteopontin levels were observed in patients with pancreatic adenocarcinoma. In addition, we also calculated osteonectin/osteopontin ratios, which were significantly (around 50%) lower in patients with pancreatic cancer when compared to those of healthy individuals (Figure 1B).

**FIGURE 2:**
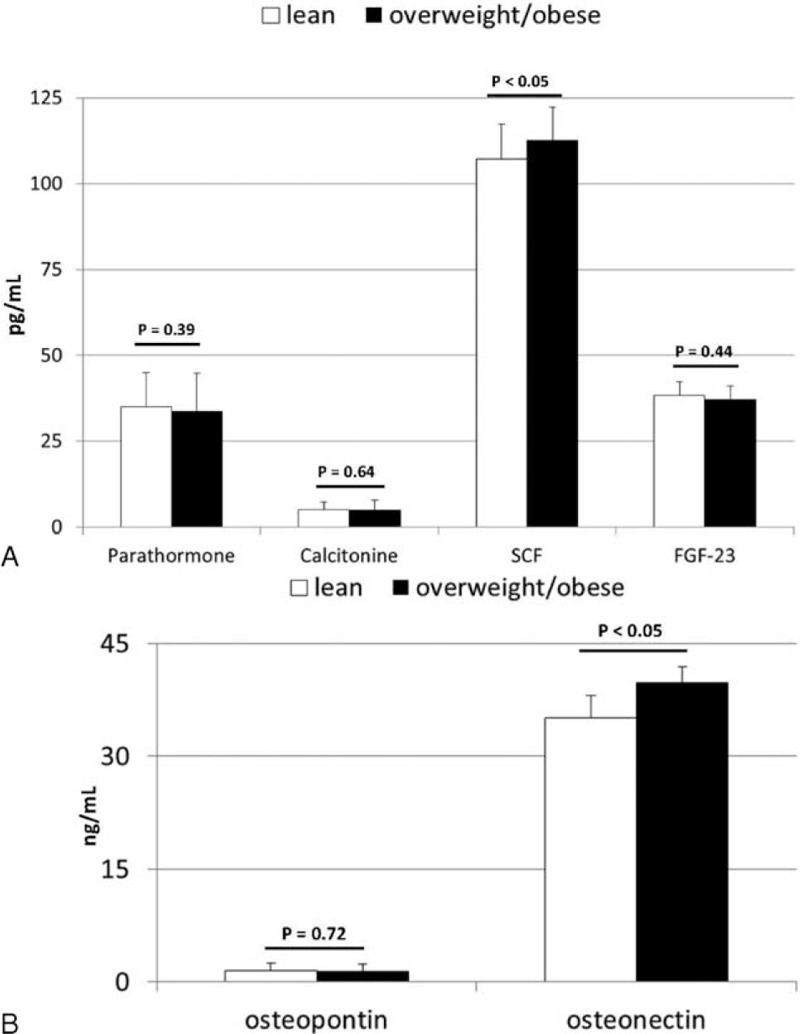
(A and B) Mean concentrations of examined biochemical factors in generally healthy individuals divided into lean and overweight/obese subgroups, together with their statistical comparison (data presented as means ± SD). SCF = stem cell factor, FGF-23 = fibroblast growth factor-23, *P* = level of significance.

In order to establish potential associations between body mass and systemic levels of the examined molecules, correlation analyses were performed. In our study, the BMI of control individuals significantly correlated with systemic levels of osteonectin and SCF (r = 0.57 and r = 0.50, respectively; *P* < 0.05 for both). We additionally subdivided our control individuals into lean and overweight/obese subgroups, according to their BMI values, and compared mean systemic levels of these biochemical substances between both subgroups. Our results revealed no significant differences in the levels for most of the examined factors. However, significantly higher SCF and osteonectin concentrations were observed in overweight/obese individuals when compared to those of lean subjects (depicted in Figure 2A and B). Interestingly, in patients with pancreatic cancer, on the other hand, no significant association between BMI and direct mean levels of the examined substances was observed, except for the osteonectin/osteopontin ratio, which was strongly associated with BMI (r = 0.52, *P* < 0.05).

### Body Mass, Examined Molecules, and Circulating BMSC

Based on these results, we investigated whether there might be a significant association between the body mass and systemic peripheral trafficking of various BMSC populations in both healthy individuals and patients with pancreatic cancer. Therefore, we selected individuals for whom we previously measured BMSC systemic circulation^22^ and subdivided them into 2 groups, lean and overweight/obese based on their BMI. In overweight/obese healthy controls, significantly higher numbers of BM-derived Lin-CD45 + CD133 + hematopoietic stem/progenitor cells (HSPCs) circulated in the peripheral blood when compared to that of lean individuals. The absolute number for other populations of circulating BMSCs did not significantly differ between healthy and overweight/obese volunteers (illustrated in Supplementary Figure 1 http://links.lww.com/MD/A361). Our results also demonstrated that the BMI of generally healthy individuals significantly correlated with the absolute number of circulating Lin-CD45 + CD133 + HSPC (r = 0.60; *P* < 0.04). Moreover, correlation analysis between the absolute number of circulating BMSC and levels of examined substances highlighted that, in generally healthy people, steady-state peripheral trafficking of HSPC and mesenchymal stem cells (MSC) is associated with systemic levels of selected biochemical molecules examined in this study, especially osteonectin (Table 2).

**TABLE 2:**
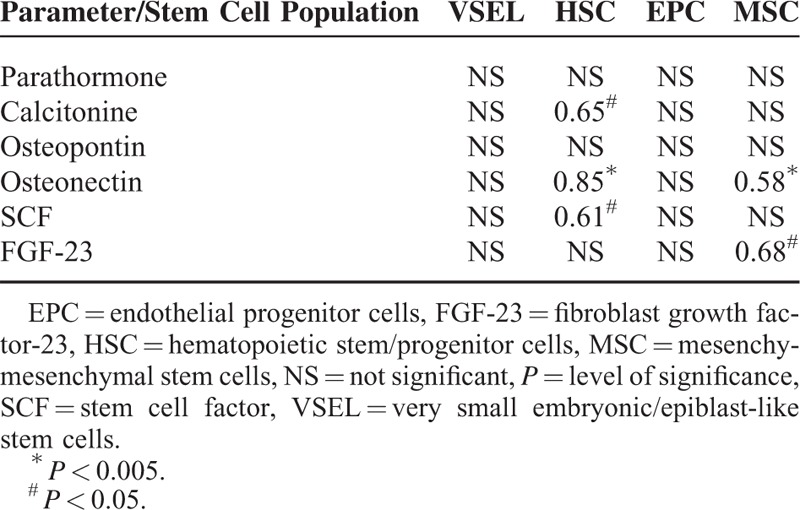
Coefficients of Correlations Between Absolute Numbers of Circulating Stem Cells’ Populations and Systemic Levels of Examined Substances Measured in Generally Healthy Individuals

Interestingly, in our study, the absolute number of circulating BMSC populations in patients with pancreatic adenocarcinoma did not significantly correlate with BMI values (r = 0.04 for very small embryonic/epiblast-like stem cells—VSEL, r = 0.12 for MSC, r = 0.17 for HSPC, and r = 0.19 for endothelial progenitor cells—EPC; in all cases *P* > 0.1). No significant difference in terms of absolute BMSC number was found between lean and overweight/obese patients with cancer in all cases (*P* > 0.09). Nevertheless, we found several associations between the examined biochemical factors and the number of circulating stem cells. The osteonectin/osteopontin coefficient was strongly associated with intensified systemic trafficking of MSC (r = 0.49, *P* < 0.05) and EPC (r = 0.55, *P* = 0.04). SCF levels, on the other hand, negatively correlated with the absolute number of both MSC (r = −0.69, *P* < 0.006) and HSPCs (r = −0.59, *P* < 0.03) in patients with cancer.

### Examined Molecules in Different AT Depots in Humans

**FIGURE 3:**
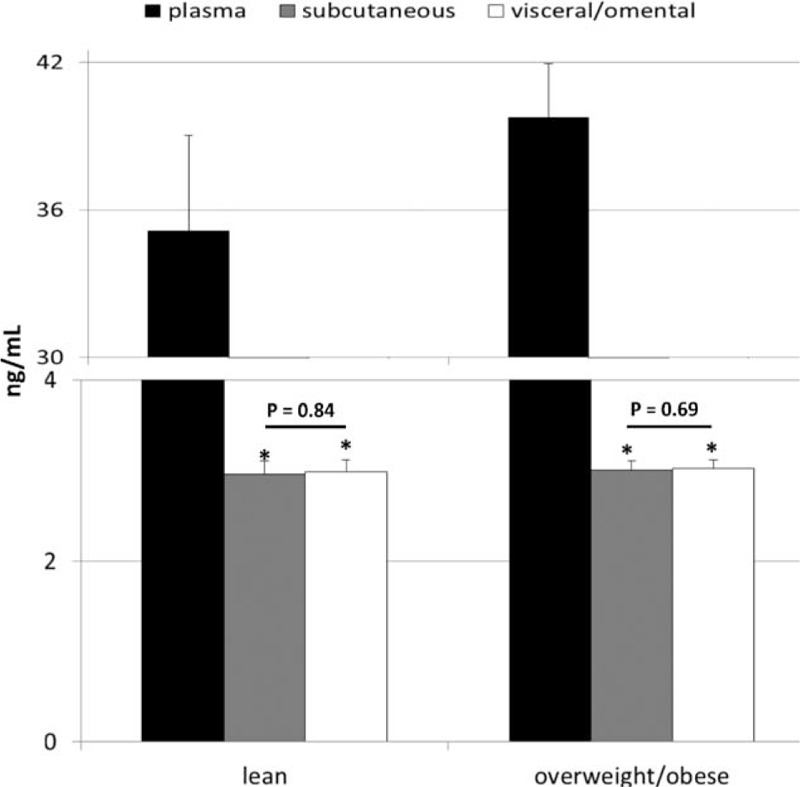
Mean values of plasma and adipose tissue levels of osteonectin in lean and overweight/obese individuals, and their statistical comparison between examined groups (data presented as means ± SD). *P* = level of significance, ∗*P* < 0.001 (vs. appropriate plasma level).

Based on these findings, we analyzed the levels of these crucial substances in subcutaneous and visceral/omental AT, and compared them to those measured in the plasma. Our analyses demonstrated that the range of both subcutaneous and visceral/omental AT levels of these substances was identical to those measured in plasma in all cases (*P* > 0.30), except for osteonectin. AT osteonectin concentrations were significantly (∼10 times) lower within AT depots than in the peripheral blood (Figure 3), however, no significant differences in its levels were observed between lean and overweight/obese individuals (*P* = 0.84 for subcutaneous and *P* = 0.32 for visceral depots). Moreover, no significant depot-specific differences were observed in AT levels in relation to all examined biochemical substances in all cases (*P* > 0.1).

## DISCUSSION

Recent clinical reports revealed a strong association between excessive AT accumulation and the development of malignancies in humans. So far, several molecular mechanisms have been proposed to be responsible for this association, including abnormal insulin/insulin-like growth factors signaling, hormonal imbalance, and modulation of the immune system functions.^2,4,12,13^ Nevertheless, growing clinical evidence outlined some weaknesses in these explanations and led to the identification of other pathways/substances that might be involved into the development of cancers among obese individuals. Such contributors may involve substances regulating the bone (marrow) homeostasis. The bone is one of the primary sites of metastasis. Thus, hormones regulating bone homeostasis seem to influence BMSC egress from the bone marrow and BMSC are known to be involved in the pathogenesis of obesity-associated cancers in humans.^17,22,32–35^ These facts shed a new light and inspire further interests in the function of the “bone–fat axis,” especially in patients with neoplasms, as interaction between these tissues may indeed be an overlooked player in cancer development/progression in humans. Therefore, we conducted a translational “step-by-step” approach to examine selected aspects of the “bone–fat axis” function in both healthy individuals and patients with pancreatic cancer.

First, we found that, in generally healthy individuals that are overweight or obese, significantly higher absolute numbers of selected BMSC populations are circulating in the peripheral blood. These are associated with body mass values and correlate with increased levels of osteonectin and/or SCF. Therefore, our results confirm previous observations that reported a slightly intensified steady-state systemic trafficking of various stem/progenitor cells in humans with excessive accumulation of AT.^36,37^ However, our findings expand the current reflection on this phenomenon and highlight that this effect seems to be mediated via the regulation of systemic levels of osteonectin and/or SCF, which are strongly associated with body mass. These 2 substances are known to significantly influence the bone, BMSC, and AT homeostasis. For example, in human AT, osteonectin regulates adipocyte differentiation and AT hyperplasia, whereas SCF regulates mitochondrial function and energy expenditure.^38–40^ Within the bone tissue, both of these substances influence osteoblasts homeostasis, bone remodeling, and maintenance of bone mass.^41–43^ Interestingly, osteonectin together with SCF are also regarded as very potent regulators of BMSC homeostasis.^44–48^ Altogether, these previous molecular reports and our clinical findings clearly highlight osteonectin (together with SCF) as a probable communication signal in the cross-talk between the bone (marrow) and AT in generally healthy individuals.

The hypothesis that osteonectin (together with SCF) may play a significant role in the metabolic communication between the bone and AT may be further supported by our results derived from the analysis of patients with pancreatic cancer. In our study, no significant association between body mass and peripheral trafficking of BMSC was observed in obesity-associated cancer patients. However, in patients with malignancies, absolute numbers of circulating BMSC were related to the biochemical imbalance in osteopontin and osteonectin (expressed in our study as osteonectin/osteopontin ratio), which, interestingly, correlated with body mass values. According to previous studies, such altered systemic biochemical balance observed in patients with cancer seems to not only create a very promising environment for systemic spread and metastasis formation by pancreatic cancer cells, but also may influence the overall prognosis.^49–53^ Our clinical analysis also demonstrates that systemic imbalance in osteopontin and osteonectin levels in humans is strongly associated with both the body mass of patients with cancer and with the peripheral circulation of MSC and EPC, which are known to significantly contribute to systemic spread of gastrointestinal tumors.^54–56^ These results support and potentiate our concept that osteonectin may be an important communication signal in the cross-talk between the bone (marrow) and AT not only in healthy individuals, but also in patients with malignancies. Nevertheless, our results are based on a relatively small population of patients, and should be confirmed in other clinical and experimental studies.

Unlike in healthy individuals, in patients with cancer, body mass did not associate with direct systemic levels of osteonectin or osteopontin, but only with the osteonectin/osteopontin ratio, which values were significantly lower in patients with pancreatic cancer compared to those of healthy controls. We hypothesize that this may be caused by the fact that, besides adipocytes, various types of (malignant) cells may contribute to both local and systemic generation of these molecules in patients with pancreatic cancer. Relatively high expression of genes responsible for osteopontin and/or osteonectin synthesis and for their receptors has already been observed in fibroblasts, the extracellular matrix surrounding (metastatic) cancer cells, and in macrophages.^53,57^ Upon cancer development/progression, systemic expression/synthesis of these molecules may not directly depend on the metabolic status anymore, but rather be influenced by malignant cells and their metabolic activity, which generally is much more rapid than that of healthy cells. Moreover, one cannot exclude the result of intensified action of these molecules and use by cells, which are expressed in several tissues by extensive fibrosis.^58–60^ Such a desmoplastic fibrotic environment is clearly observed in patients with pancreatic cancer both at the molecular and clinical levels and is often regarded as one of the most crucial factors responsible for the wide and rapid spread of this disease. Also, it creates a limitation that prohibits successful surgical and pharmacological intervention in affected individuals. Nevertheless, this aspect requires further exploration especially in experimental studies.

Finally, our study also demonstrated that range of levels of almost all examined molecules were comparable between the plasma and AT environment, except for osteonectin level, which was significantly (around 10 times) lower within AT depots. Previous reports demonstrated that, in (morbidly) obese patients, significantly higher expression of osteonectin is observed in the AT in comparison to lean individuals.^60^ However, our study demonstrates that being slightly overweight/obese is not associated with significant changes in osteonectin AT levels, but rather translates “only” in increased peripheral concentrations of this molecule in humans. These results were also in agreement with our previous findings regarding the molecular composition of different depots of human AT, in which we described several differences between human plasma and AT-derived FIF, yet excess fat accumulation in humans was not associated with major changes in its biochemical composition.^24,26,29^

In summary, our study delivered novel translational information about functions of the “bone–fat axis” in humans. Foremost, it highlighted osteonectin, osteopontin, and SCF as probable communication signals between the bone and AT in humans and described their associations with BMSC systemic trafficking in both healthy individuals and patients with pancreatic cancer. We postulate that these molecules may be overlooked biochemical players, linking obesity and BMSCs with cancer development/progression in humans. Further experimental and in vivo studies are necessary to confirm these clinical observations and to precisely define the mechanisms by which these molecules influence both BMSC homeostasis and its relation to AT (patho)physiology in humans.
